# Assessment of Blood Biomarker Profile After Acute Concussion During Combative Training Among US Military Cadets: A Prospective Study From the NCAA and US Department of Defense CARE Consortium

**DOI:** 10.1001/jamanetworkopen.2020.37731

**Published:** 2021-02-22

**Authors:** Christopher C. Giza, Michael McCrea, Daniel Huber, Kenneth L. Cameron, Megan N. Houston, Jonathan C. Jackson, Gerald McGinty, Paul Pasquina, Steven P. Broglio, Alison Brooks, John DiFiori, Stefan Duma, Jaroslaw Harezlak, Joshua Goldman, Kevin Guskiewicz, Thomas W. McAllister, David McArthur, Timothy B. Meier, Jason P. Mihalik, Lindsay D. Nelson, Steven Rowson, Jessica Gill, and the CARE Consortium Investigators, Tatiana Foroud, Barry Katz, Andrew Saykin, Darren E. Campbell, Steven Svoboda

**Affiliations:** 1 Department of Neurosurgery, UCLA Steve Tisch BrainSPORT Program, University of California, Los Angeles, Los Angeles; 2 Department of Pediatrics, UCLA Steve Tisch BrainSPORT Program, University of California, Los Angeles, Los Angeles; 3 Department of Neurosurgery, Medical College of Wisconsin, Milwaukee; 4 John A. Feagin Sports Medicine Fellowship, Keller Army Community Hospital, West Point, New York; 5 Department of Physical Medicine and Rehabilitation, Uniformed Services University, Bethesda, Maryland; 6 US Air Force Academy, Colorado Springs, Colorado; 7 Michigan Concussion Center, University of Michigan, Ann Arbor; 8 Department of Orthopedics and Rehabilitation, School of Medicine and Public Health, University of Wisconsin, Madison; 9 Hospital for Special Surgery, New York, New York; 10 Department of Biomedical Engineering, Virginia Tech, Blacksburg; 11 Department of Epidemiology and Biostatistics School of Public Health-Bloomington, Indiana University, Bloomington; 12 Department of Family Medicine, UCLA Steve Tisch BrainSPORT Program, University of California, Los Angeles, Los Angeles; 13 Matthew Gfeller Sport-Related Traumatic Brain Injury Research Center, Department of Exercise and Sport Science, University of North Carolina at Chapel Hill, Chapel Hill; 14 Department of Psychiatry, Indiana University School of Medicine, Indianapolis; 15 National Institute of Nursing Research, National Institutes of Health, Bethesda, Maryland; 16 US Military Academy at West Point, New York

## Abstract

**Importance:**

Validation of protein biomarkers for concussion diagnosis and management in military combative training is important, as these injuries occur outside of traditional health care settings and are generally difficult to diagnose.

**Objective:**

To investigate acute blood protein levels in military cadets after combative training–associated concussions.

**Design, Setting, and Participants:**

This multicenter prospective case-control study was part of a larger cohort study conducted by the National Collegiate Athletic Association and the US Department of Defense Concussion Assessment Research and Education (CARE) Consortium from February 20, 2015, to May 31, 2018. The study was performed among cadets from 2 CARE Consortium Advanced Research Core sites: the US Military Academy at West Point and the US Air Force Academy. Cadets who incurred concussions during combative training (concussion group) were compared with cadets who participated in the same combative training exercises but did not incur concussions (contact-control group). Clinical measures and blood sample collection occurred at baseline, the acute postinjury point (<6 hours), the 24- to 48-hour postinjury point, the asymptomatic postinjury point (defined as the point at which the cadet reported being asymptomatic and began the return-to-activity protocol), and 7 days after return to activity. Biomarker levels and estimated mean differences in biomarker levels were natural log (ln) transformed to decrease the skewness of their distributions. Data were collected from August 1, 2016, to May 31, 2018, and analyses were conducted from March 1, 2019, to January 14, 2020.

**Exposure:**

Concussion incurred during combative training.

**Main Outcomes and Measures:**

Proteins examined included glial fibrillary acidic protein, ubiquitin C-terminal hydrolase-L1, neurofilament light chain, and tau. Quantification was conducted using a multiplex assay (Simoa; Quanterix Corp). Clinical measures included the Sport Concussion Assessment Tool–Third Edition symptom severity evaluation, the Standardized Assessment of Concussion, the Balance Error Scoring System, and the 18-item Brief Symptom Inventory.

**Results:**

Among 103 military service academy cadets, 67 cadets incurred concussions during combative training, and 36 matched cadets who engaged in the same training exercises did not incur concussions. The mean (SD) age of cadets in the concussion group was 18.6 (1.3) years, and 40 cadets (59.7%) were male. The mean (SD) age of matched cadets in the contact-control group was 19.5 (1.3) years, and 25 cadets (69.4%) were male. Compared with cadets in the contact-control group, those in the concussion group had significant increases in glial fibrillary acidic protein (mean difference in ln values, 0.34; 95% CI, 0.18-0.50; *P* < .001) and ubiquitin C-terminal hydrolase-L1 (mean difference in ln values, 0.97; 95% CI, 0.44-1.50; *P* < .001) levels at the acute postinjury point. The glial fibrillary acidic protein level remained high in the concussion group compared with the contact-control group at the 24- to 48-hour postinjury point (mean difference in ln values, 0.22; 95% CI, 0.06-0.38; *P* = .007) and the asymptomatic postinjury point (mean difference in ln values, 0.21; 95% CI, 0.05-0.36; *P* = .01). The area under the curve for all biomarkers combined, which was used to differentiate cadets in the concussion and contact-control groups, was 0.80 (95% CI, 0.68-0.93; *P* < .001) at the acute postinjury point.

**Conclusions and Relevance:**

This study’s findings indicate that blood biomarkers have potential for use as research tools to better understand the pathobiological changes associated with concussion and to assist with injury identification and recovery from combative training–associated concussions among military service academy cadets. These results extend the previous findings of studies of collegiate athletes with sport-associated concussions.

## Key Points

**Question:**

Is concussion incurred during combative training associated with changes in blood levels of protein biomarkers for traumatic brain injury among military service academy cadets?

**Findings:**

In this case-control study of 103 military service academy cadets in the US, the levels of glial fibrillary acidic protein and ubiquitin C-terminal hydrolase-L1 biomarkers among cadets who incurred concussions during combative training indicated significant increases compared with baseline values and values found in matched cadets who participated in the same combative training exercises but did not incur concussions. Neurofilament light chain levels were significantly higher among cadets with concussions compared with those without concussions at less than 6 hours after injury to 7 days after return to activity.

**Meaning:**

In this study, blood biomarkers delineated physiological changes after concussions that were incurred during military combative training, indicating patterns that were largely consistent with those reported in studies of sport-associated concussions.

## Teaser

This case-control study examines whether blood levels of protein biomarkers for traumatic brain injury change after concussions incurred during combative training exercises among US military service academy cadets.

## Introduction

Mild traumatic brain injuries (TBIs) or concussions are a substantial public health problem, occurring with peak incidence in adolescence and young adulthood.^1^ Common mechanisms for concussion in this age range are typically associated with sports and other recreational activities.^2^ Although several studies have examined acute injury response and recovery from sport-associated concussions, recreational and occupational injuries have been less characterized. Military service academy (MSA) cadets represent a group of young, healthy individuals who have a higher risk of incurring mild TBI in the setting of combative training. Cadets are required to participate in combative training exercises, which, in the context of this study, were defined to include boxing, self-defense, martial arts, and other military training, all of which may result in head impacts. It is essential that studies of combative training–associated concussions have an active control group of cadets who are undergoing the same combative training exercises but have not incurred concussions (defined as a contact-control group). Symptom burdens associated with the stress of combative training may overlap with those of concussion, and the potential for incurring subconcussive impacts as part of combative training needs to also be considered.^3^ Data also indicate that aerobic activity in the absence of head injuries has implications for blood biomarkers of concussion.^4,5^

Blood biomarkers have been increasingly studied in military settings because acute clinical assessment of concussion may be challenging under the conditions of both military training and combat deployment.^6,7^ Conflicting sources of symptoms (eg, pain and anxiety) can confound symptom-based concussion assessment tools. For cadets, concerns about the career implications of a concussion diagnosis may produce underreporting of symptoms, underdiagnosis, and premature return to activity and duty. Active-duty personnel’s desire to return to their units may similarly complicate accurate concussion diagnosis and return-to-duty dispositions. Quantitative biological measures to inform concussion diagnosis have important implications in the military that may exceed their role in conventional civilian settings.

Blood-based biomarkers offer the potential for objective detection of molecules associated with the underlying pathophysiologic changes associated with concussion.^8^ Glial fibrillary acidic protein (GFAP) and ubiquitin C-terminal hydrolase-L1 (UCH-L1) levels have been associated with positive computed tomographic findings in patients presenting to the emergency department with mild TBIs.^9,10^ Higher levels of GFAP have also been reported in patients with mild TBI and positive magnetic resonance imaging findings.^11,12^ Increased neurofilament light chain (NF-L) levels have been associated with TBI, concussion, and persistent postconcussive symptoms as well as other neurological disorders in the absence of TBI.^13,14^ Increased tau levels have been implicated in repetitive mild TBI, but study results have been mixed.^15,16,17^

This study targeted concussions that occurred in combative training among MSA cadets to assess whether blood-based biomarkers (GFAP, UCH-L1, NF-L, and tau) indicated patterns similar to those observed after sport-associated concussions or mild TBI. The overarching hypothesis was that combative training–associated concussions would be associated with increased brain injury–associated proteins in the blood and that these biomarkers could enhance the accurate detection of concussion and recovery among MSA cadets.

## Methods

### Study Design and Population

This study was part of a larger multicenter prospective cohort study conducted by the Advanced Research Core within the National Collegiate Athletic Association and the US Department of Defense Concussion Assessment Research and Education (CARE) Consortium^18^ from February 20, 2015, to May 31, 2018. Data for the present cross-sectional study were collected from August 1, 2016, to May 31, 2018. The study was approved by the institutional review board of the Medical College of Wisconsin and the Human Research Protection Office. Written informed consent was obtained from all participants. All data were deidentified. Cadets were recruited from the US Military Academy at West Point and the US Air Force Academy. This study followed the Strengthening the Reporting of Observational Studies in Epidemiology (STROBE) guideline for case-control studies.^19^

A total of 1178 MSA cadets completed baseline clinical assessments, with 1085 cadets also providing a blood sample at baseline. Cadets who incurred concussions in the setting of combative training (n = 67; concussion group) were matched on a 2:1 ratio (based on institution, sex, and sport category) with cadets who participated in the same combative training exercises but did not incur concussions (n = 36; contact-control group). Sport categories were football, hockey, soccer, and other. Concussion was defined using the consensus definition from the US Department of Defense evidence-based guidelines.^20^ Both groups underwent the same protocols for baseline and serial assessment, including clinical measures and blood sample collection.

### Procedures, Collection, and Bioassays

The CARE Advanced Research Core protocol involved preseason baseline clinical testing and blood sample collection. The CARE Advanced Research Core postinjury protocol included collection of clinical data and blood samples from cadets with concussions at the following assessment points: (1) acute postinjury (<6 hours); (2) 24 to 48 hours postinjury; (3) asymptomatic postinjury defined as the point at which the cadet reported being asymptomatic and the return-to-activity (RTA) protocol was initiated; and (4) 7 days after unrestricted RTA (7-day post-RTA). Not all participants completed all visits.

Clinical data obtained at all postinjury assessment points included the Sport Concussion Assessment Tool–Third Edition (SCAT-3) symptom severity evaluation (symptom severity score range, 0-132, with higher scores indicating more severe symptoms), the Standardized Assessment of Concussion (SAC; score range, 0-30, with higher scores indicating better cognitive functioning), and the Balance Error Scoring System (BESS; score range, 0-60, with higher scores indicating worse static postural stability). The 18-item Brief Symptom Inventory (BSI-18; score range, 0-72, with higher scores indicating higher levels of psychological distress) was obtained at all assessment points with the exception of the acute postinjury visit.

Nonfasting blood samples were collected using standard venipuncture at baseline and at all postinjury assessment points in a 10-mL red-top tube for serum and allowed to clot for 30 minutes. Samples were then centrifuged within 60 minutes of blood collection at 1500*g* for 15 minutes. Next, they were aliquoted into cryovials, frozen at −80 °C, and shipped on dry ice to the CARE Consortium biorepository at the Indiana University School of Medicine National Institute of Neurological Disorders and Stroke BioSEND Core. Samples were then sent to the National Institutes of Health for analysis.

Biomarker levels were assayed using single-protein array technology (Simoa; Quanterix Corp) in simultaneous multiplex assays to quantify GFAP, UCH-L1, NF-L, and tau levels (pg/mL). Assays were batched to minimize variability, with each batch run with appropriate standards and controls to ensure reliability. Groups were distributed randomly across plates, and longitudinal samples from the same individual were run on the same plate to reduce potential batch effects. All samples were analyzed in duplicate, and the technicians running the assays were blinded to all data. In rare instances in which coefficients of variance exceeded 20%, samples were rerun. Data were not used if either intra-assay or interassay performance was higher than 20%, which occurred in less than 5% of samples. The lower limits of quantification were 0.241 pg/mL for NF-L, 0.467 pg/mL for GFAP, 5.45 pg/mL for UCH-L1, and 0.053 pg/mL for tau.

### Statistical Analysis

Statistical analyses were performed using IBM SPSS Statistics, version 24.0 (IBM Corp). Demographic characteristics and clinical outcome group comparisons at baseline were evaluated using *t* tests and χ^2^ or Fisher exact tests. Biomarker levels and estimated mean differences in biomarker levels were natural log (ln) transformed to decrease the skewness of their distributions. Linear mixed-effects models were used to evaluate changes in biomarker levels and to examine the results of clinical assessments (ie, SCAT-3, SAC, BESS, and BSI-18 scores) within cadets over time as a function of group, with visit modeled as a repeated factor (ie, baseline, acute postinjury, 24-48 hours postinjury, start of RTA protocol, and 7 days post-RTA), group (ie, concussion and contact-control), and group-by-visit interaction. Estimated mean differences and 95% CIs were provided for linear mixed-effects post hoc analyses. Bonferroni correction to protect the familywise error at α = .05 was conducted for post hoc tests, when indicated. Sensitivity analyses were performed to assess whether acute injury severity (based on the SCAT-3 symptom severity score)differed significantly between cadets in the concussion group who did and did not have blood samples collected at the acute postinjury point. Pearson correlation coefficients were used to examine the association between biomarkers and clinical outcome measures.

Receiver operating characteristic curves and area under the curve (AUC) with 95% CIs were used to quantify the ability of markers to discriminate cadets with concussion and those without concussion at the acute and 24- to 48-hour postinjury points. Logistic regression analyses were conducted to examine whether the inclusion of blood biomarkers was significantly associated with greater discrimination of cadets in the concussion group vs the contact-control group compared with self-reported symptoms alone. Tests were 2-sided with a significance threshold of *P* < .05. Data were analyzed from March 1, 2019, to January 14, 2020.

## Results

###  Demographic Characteristics

Of the 1178 MSA cadets with full clinical baseline data and blood samples, 217 cadets incurred a concussion; of those, 210 cadets had blood samples collected after injury. A total of 77 out-of-sport concussions (defined as concussions incurred outside of varsity, organized, or intramural sports) were identified. Two cadets each had 2 concussions during the study period, and only the first concussion was included in our analysis. Out-of-sport concussions that were not associated with combative training were excluded (1 concussion was associated with snowboarding, 6 concussions were associated with unorganized sports, and the cause of 1 concussion was undetermined). These exclusions produced a final sample of 103 cadets; of those, 67 cadets were in the concussion group and 36 matched cadets were in the contact-control group. The mean (SD) age of cadets in the concussion group was 18.6 (1.3) years, and 40 cadets (59.7%) were male. The mean (SD) age of matched cadets in the contact-control group was 19.5 (1.3) years, and 25 cadets (69.4%) were male. Most cadets in the concussion group (55 cadets [82.1%]) were injured while boxing. Eight cadets (11.9%) incurred concussions during other combative sports, 3 cadets (4.5%) incurred concussions during military training, and 1 cadet (1.5%) incurred a concussion during judo (Table).

**Table.** Participant Characteristics

Characteristic	No. (%)		Test statistic	*P* value
	Concussion group (n = 67)	Contact-control group (n = 36)		
Age, mean (SD), y	18.6 (1.3)	19.5 (1.3)	*t* = −3.27	.001
Male sex	40 (59.7)	25 (69.4)	χ^2^ = 0.955	.33
Height, mean (SD), cm	173.93 (9.87)	178.26 (8.41)	*t* = −2.22	.02
Weight, mean (SD), kg	73.85 (15.94)	80.42 (14.73)	*t* = −2.04	.04
Years of sport participation, mean (SD)	9.04 (3.98)	10.52 (5.49)	*t* = −1.09	.28
Previous concussions, No.				
0	49 (73.1)	26 (72.2)	Fisher exact^a^	.30
1	16 (23.9)	8 (22.2)		
2	2 (3.0)	0		
≥3	0	2 (5.6)		
SCAT-3 symptom severity score, mean (SD)	10.83 (14.97)	4.11 (7.86)	*t* = 2.96	.004
SAC total score, mean (SD)	28.17 (1.59)	28.54 (1.48)	*t* = −1.12	.26
BESS total score, mean (SD)	17.11 (6.41)	14.64 (6.41)	*t* = 1.84	.07
BSI-18 global severity index raw score, mean (SD)	4.94 (9.06)	1.37 (2.66)	*t* = 2.97	.004
Race				
White	45 (67.2)	27 (75.0)	Fisher exact^a^	.36
Black	7 (10.4)	5 (13.9)		
Other, unknown, or not reported	15 (22.4)	4 (11.1)		
Ethnicity				
Non-Hispanic	59 (88.1)	32 (88.9)	Fisher exact^a^	.70
Hispanic	4 (6.0)	3 (8.3)		
Unknown or not reported	4 (6.0)	1 (2.8)		
ADHD	1 (1.5)	2 (5.6)	Fisher exact^a^	.28
Sport at enrollment				
Nonathlete	41 (61.2)	14 (38.9)	Fisher exact^a^	.01
Baseball	1 (1.5)	0		
Cross-country or track	3 (4.5)	0		
Cheerleading	1 (1.5)	0		
Fencing	1 (1.5)	0		
Field event	1 (1.5)	0		
Football	5 (7.5)	7 (19.4)		
Gymnastics	4 (6.0)	0		
Ice hockey	0	2 (5.6)		
Lacrosse	1 (1.5)	2 (5.6)		
Rugby	2 (3.0)	5 (13.9)		
Soccer	2 (3.0)	4 (11.1)		
Swimming	1 (1.5)	0		
Volleyball	2 (3.0)	0		
Water polo	1 (1.5)	0		
Wrestling	1 (1.5)	2 (5.6)		
Injury characteristics				
Injury activity				
Boxing	55 (82.1)	NA	NA	NA
Combative exercise	8 (11.9)			
Judo	1 (1.5)			
Other military training or movement	3 (4.5)			
Time to symptom-free, mean (SD), d	14.27 (12.90)	NA	NA	NA
Time lost from participation, mean (SD), d	24.10 (19.74)	NA	NA	NA
Loss of consciousness	2 (3.0)	NA	NA	NA
Posttraumatic amnesia	6 (9.0)	NA	NA	NA
Retrograde amnesia	5 (7.5)	NA	NA	NA
Hours to postinjury blood sample collection, median (IQR)	2.00 (1.40-4.53)	NA	NA	NA
Hours to 24-hour blood sample collection, median (IQR)	40.65 (25.33-65.15)	NA	NA	NA

Abbreviations: ADHD, attention-deficit/hyperactivity disorder; BESS, Balance Error Scoring System (score range, 0 to 60, with higher scores indicating worse static postural stability); BSI-18, 18-item Brief Symptom Inventory (score range, 0 to 72, with higher scores indicating higher levels of psychological distress); IQR, interquartile range; NA, not applicable; SAC, Standardized Assessment of Concussion (score range, 0 to 30, with higher scores indicating better cognitive functioning); SCAT-3, Sport Concussion Assessment Tool–Third Edition (symptom severity score range, 0 to 132, with higher scores indicating more severe symptoms).

^a^ A Fisher exact test was used for comparisons with counts less than 5.

No statistically significant differences in sex, years of sport participation, number of previous concussions, race, or ethnicity were found between groups. Cadets in the concussion group were significantly younger (mean difference, −0.87; 95% CI, −1.40 to −0.34; *P* = .001), of shorter height (mean difference, −4.33; 95% CI, −8.19 to −0.47; *P* = .03), and of lower weight (mean difference, −6.57; 95% CI, −12.95 to −0.19; *P* = .04) than those in the contact-control group but did not significantly differ in body mass index (calculated as weight in kilograms divided by height in meters squared; mean difference, −0.92; 95% CI, −2.29 to 0.44; *P* = .18). Although there were no statistically significant group differences in SAC or BESS scores at baseline, cadets in the concussion group had higher baseline SCAT-3 symptom severity scores (mean difference, 6.89; 95% CI, 2.34-11.43; *P* = .003) and higher BSI-18 global severity scores (mean difference, 3.61; 95% CI, 1.19-6.04; *P* = .004) than those in the contact-control group. Descriptive statistics for clinical outcome assessments at baseline and all postinjury points are provided in eTable 1 in the Supplement.

### Clinical Measures

For clinical measures, the main effects of group (concussion vs contact-control) were observed in the SCAT-3 symptom severity score (*F*_1,91.1_ = 55.6; *P* < .001), the BESS score (*F*_1,101.4_ = 15.2; *P* < .001), and the BSI-18 global severity score (*F*_1,83_ = 9.8; *P* = .002), with the concussion group having higher SCAT-3 symptom severity scores (mean difference, 11.16; 95% CI, 8.19-14.13), higher BSI-18 global severity scores (mean difference, 2.21; 95% CI, 0.81-3.62), and worse performance on the BESS (mean difference, 4.04; 95% CI, 1.99-6.10). The SAC results indicated a group main effect that was not statistically significant (mean difference, −0.37; 95% CI, −0.76 to 0.02).

Statistically significant interactions were found between group and time for the SCAT-3 (*F*_4,352.9_ = 35.4; *P* < .001), the SAC (*F*_4,355.7_ = 2.5; *P* = .04), and the BESS (*F*_4,355.5_ = 3.8; *P* = .005). The BSI-18 group and time interaction was not statistically significant (*F*_3,262.9_ = 2.3; *P* = .07).

**Figure 1.**
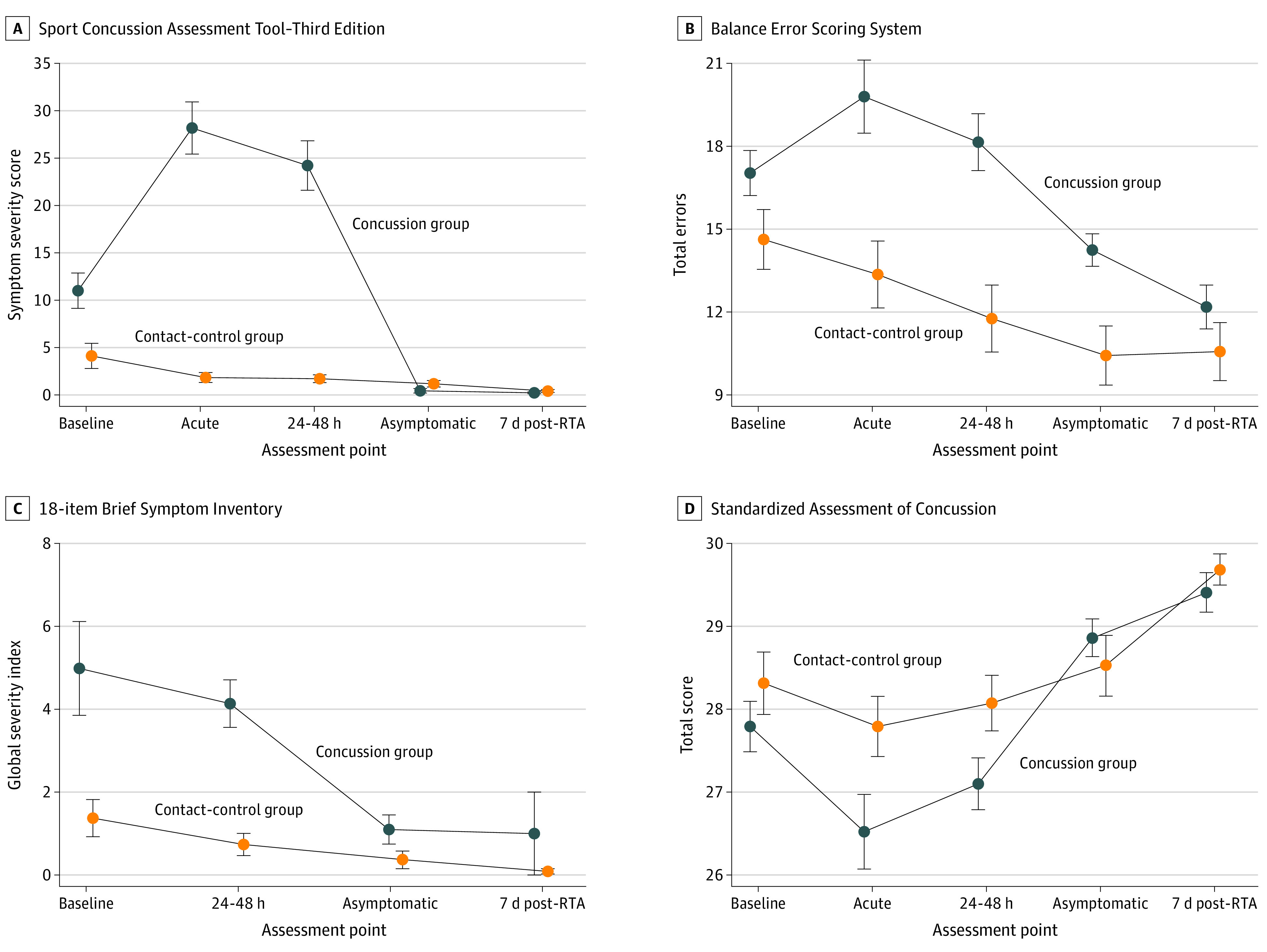
Baseline and Postinjury Assessment Data for Cadets With and Without Concussions The concussion group includes cadets who incurred concussions during combative training, and the contact-control group includes matched cadets who participated in the same combative training but did not incur concussions. Acute indicates the acute postinjury (<6 hours) point; 24-48 h, the 24- to 48-hour postinjury point; asymptomatic, the postinjury point at which the cadet reported being asymptomatic and return-to-activity (RTA) protocol was initiated; and 7d post-RTA, 7 days after unrestricted return to activity. Error bars represent plus 1 and minus 1 SE. A, Symptom severity score range, 0 to 132, with higher scores indicating more severe symptoms. B, Score range, 0 to 60, with higher scores indicating worse static postural stability. C, Score range, 0 to 72, with higher scores indicating higher levels of psychological distress. D, Score range, 0 to 30, with higher scores indicating better cognitive functioning.

Post hoc analyses indicated that the concussion group had significantly increased SCAT-3 symptom severity scores (at baseline, mean difference, 6.92 [95% CI, 2.27-11.57]; *P* = .004; at acute postinjury, mean difference, 27.06 [95% CI, 22.11-32.02]; *P* < .001; and at 24-48 hours postinjury, mean difference, 22.28 [95% CI, 17.54-27.01]; *P* < .001) and worse performance on the SAC (at acute postinjury, mean difference, −0.85 [95% CI, −1.48 to −0.22]; *P* = .009 and at 24-48 hours postinjury, mean difference, −0.69 [95% CI, −1.30 to −0.08]; *P* = .03) and the BESS (at acute postinjury, mean difference, 6.22 [95% CI, 3.34-9.10]; *P* < .001 and at 24-48 hours postinjury, mean difference, 6.19 [95% CI, 3.40-8.98]; *P* < .001) compared with the contact-control group at both the acute and 24- to 48-hour postinjury points (Figure 1). Performance on the BESS remained worse among the concussion group compared with the contact-control group at the asymptomatic postinjury point (mean difference, 3.86; 95% CI, 1.11-6.60; *P* = .006). Significant pairwise comparisons of the clinical outcomes are available in eTable 2 and eTable 3 in the Supplement.

### Primary Biomarker Analysis

Results of the linear mixed-effects analysis revealed a significant main effect of group for GFAP levels (*F*_1,102_ = 9.06; *P* = .003) and a significant time by group interaction (*F*_4,283_ = 6.00; *P* = .02). A significant time by group interaction was also observed for UCHL-1 levels (*F*_4,201_ = 2.86; *P* = .02). For NF-L levels, there was a main effect of group (*F*_1,102_ = 9.03; *P* = .003) but no interaction with time. Tau levels had no significant main effects. Descriptive statistics of the raw (ie, nontransformed) biomarker values are available in eTable 4 in the Supplement. Medians for the time from injury to clinical testing and blood sample collection were 2.00 hours (interquartile range [IQR], 1.40-4.53 hours) at the acute postinjury point, 40.65 hours (IQR, 25.33-65.15 hours) at the 24- to 48-hour postinjury point, 12.00 days (IQR, 6.68-18.46 days) at the asymptomatic postinjury point, and 27.97 days (IQR, 20.86-42.96 days) at the 7-day post-RTA point.

Post hoc analyses revealed no differences between the concussion and contact-control groups in GFAP levels (mean difference in ln values, 0.188; 95% CI, −0.007 to 0.383; *P* = .06) or UCH-L1 levels (mean difference in ln values, −0.243; 95% CI, −1.005 to 0.519; *P* = .53) at baseline. The concussion group had significantly higher levels of NF-L compared with the contact-control group (mean difference in ln values, 0.309; 95% CI, 0.105-0.513; *P* = .003). The contact-control group had stable levels across time for GFAP and UCH-L1.

The GFA*P* values at the acute postinjury point were greater than those at the asymptomatic postinjury point (mean difference in ln values, 0.125; 95% CI, 0.016-0.234; *P* = .01) and the 7-day post-RTA point (mean difference in ln values, 0.177; 95% CI, 0.065-0.289; *P* < .001), and GFA*P* values at the 24- to 48-hour postinjury point were greater than those at the 7-day post-RTA point (mean difference in ln values, 0.107; 95% CI, 0.009-0.205; *P* = .02). The UCH-L1 values at the acute postinjury point were higher than those at the 24- to 48-hour postinjury point (mean difference in ln values, 0.770; 95% CI, 0.208-1.331; *P* = .001), the asymptomatic postinjury point (mean difference in ln values, 0.624; 95% CI, 0.078-1.169; *P* = .01), and the 7-day post-RTA point (mean difference in ln values, 0.870; 95% CI, 0.329-1.410; *P* < .001).

**Figure 2.**
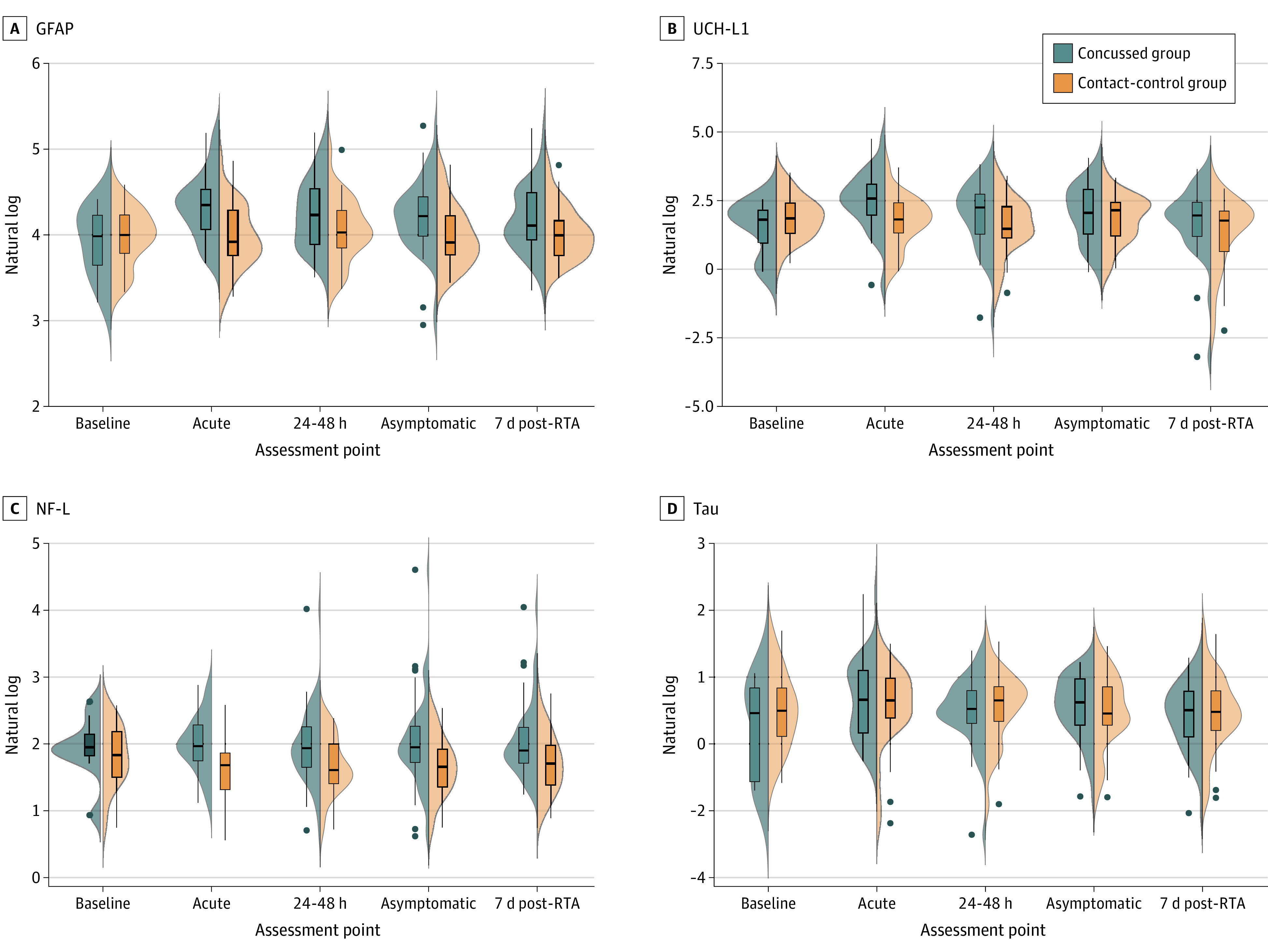
Baseline and Postinjury Biomarker Levels in Cadets With and Without Concussions The concussion group includes cadets who incurred concussions during combative training, and the contact-control group includes matched cadets who participated in the same combative training but did not incur concussions. Acute indicates the acute postinjury (<6 hours) point; 24-48 h, the 24- to 48-hour postinjury point; asymptomatic, the postinjury point at which the cadet reported being asymptomatic and return-to-activity (RTA) protocol was initiated; and 7d post-RTA, 7 days after unrestricted return to activity. Violin plots for each group illustrate the kernel probability density, with the width of the shaded area representing the proportion of the data located there. Biomarker levels represent natural log-transformed scale. Box and whisker plots are overlaid to illustrate the 5th, 25th, 50th, 75th, and 95th percentiles. Filled dots represent outlying values. GFAP indicates glial fibrillary acidic protein; NF-L, neurofilament light chain; and UCH-L1, ubiquitin C-terminal hydrolase-L1.

The full profile of biomarker levels for the concussion and contact-control groups over time is shown in Figure 2. When examining group by time interaction, cadets in the concussion group had increased GFAP levels compared with those in the contact-control group at the acute postinjury point (mean difference in ln values, 0.34; 95% CI, 0.18-0.50; *P* < .001), the 24- to 48-hour postinjury point (mean difference in ln values, 0.22; 95% CI, 0.06-0.38; *P* = .007), and the asymptomatic postinjury point (mean difference in ln values, 0.21; 95% CI, 0.05-0.36; *P* = .01). The concussion group also had increased levels of UCH-L1 compared with the contact-control group at the acute postinjury point (mean difference in ln values, 0.97; 95% CI, 0.44-1.50; *P* < .001), with no group differences at the 24- to 48-hour postinjury, asymptomatic postinjury, and 7-day post-RTA points. No group by time interactions were observed for NF-L or tau levels. Further description of the biomarker profiles between groups over time are available in the eFigure in the Supplement, and significant pairwise comparisons of biomarkers are available in eTable 5 and eTable 6 in the Supplement.

Of the 67 cadets with concussions, 7 cadets (10.4%) had blood samples collected at both baseline and acute postinjury points, and 30 cadets (44.8%) had blood samples collected at both the acute and 24- to 48-hour postinjury points. Sensitivity analyses indicated that cadets with and without blood samples collected at the acute postinjury point reported similar SCAT-3 symptom severity scores at both the acute (*t*[43] = .09; *P* = .93) and 24- to 48-hour (*t*[59] = −1.16; *P* = .25) points.

### Receiver Operating Characteristic Analysis

**Figure 3.**
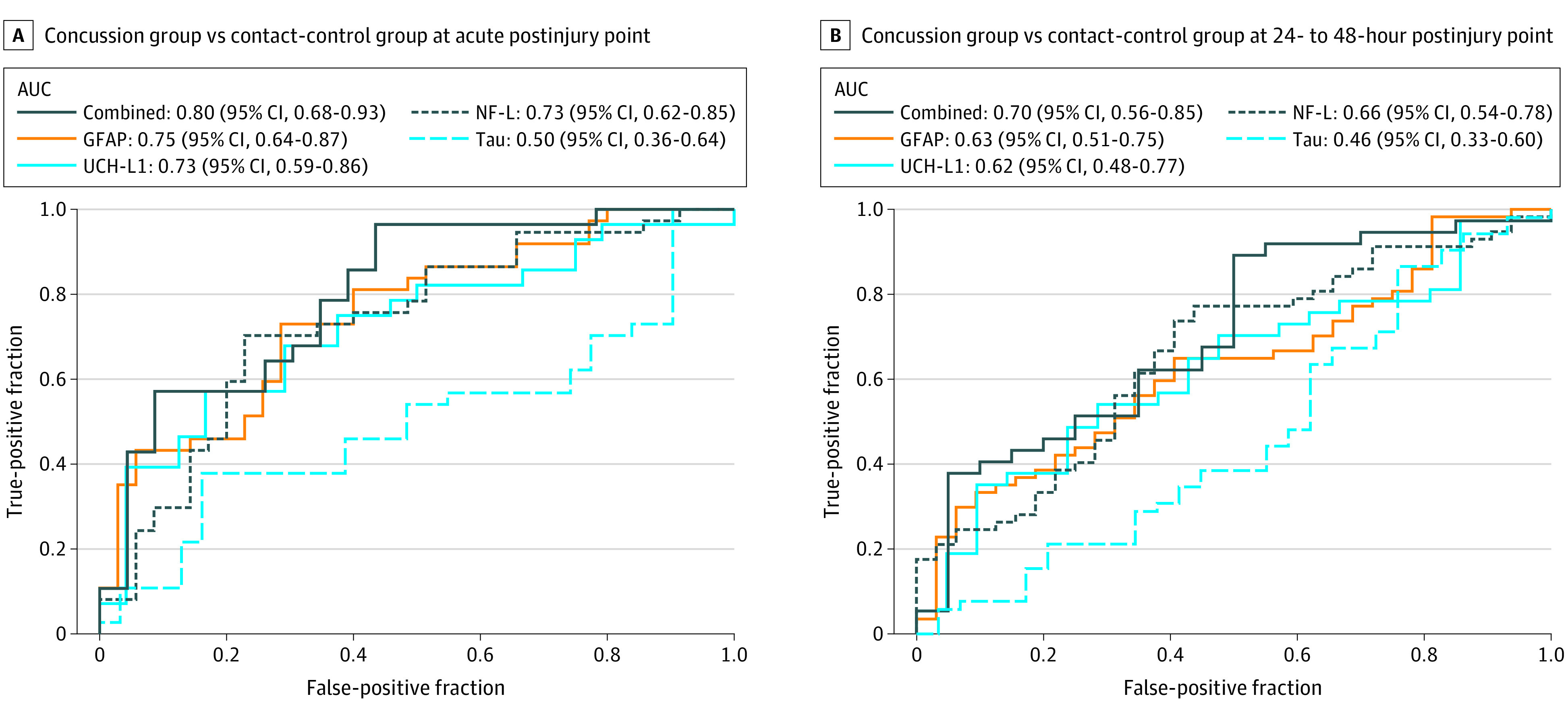
Receiver Operating Characteristic Results for Biomarkers Differentiating Cadets With and Without Concussions at Acute and 24- to 48-Hour Postinjury Points AUC indicates area under the curve; GFAP, glial fibrillary acidic protein; NF-L, neurofilament light chain; and UCH-L1, ubiquitin C-terminal hydrolase-L1.

To evaluate the ability of these biomarkers to distinguish cadets in the concussion group from those in the contact-control group, receiver operating characteristic curves and AUC analyses were conducted. Individual biomarkers were assessed at the acute and 24- to 48-hour postinjury points (Figure 3).

The GFAP and UCH-L1 biomarkers combined had an AUC of 0.78 (95% CI, 0.66-0.91; *P* < .001), and the AUC for all 4 biomarkers combined was 0.80 (95% CI, 0.68-0.93; *P* < .001) at the acute postinjury point. The AUC values were lower at the 24- to 48-hour postinjury point. Overall, the addition of blood biomarkers was not significantly associated with greater discrimination between the concussion and contact-control groups beyond the SCAT-3 results alone at either the acute or 24- to 48-hour postinjury points.

### Clinical Outcome Measures and Biomarkers

In addition, Pearson correlations were used to examine the association between biomarkers and clinical outcomes at the acute and 24- to 48-hour postinjury points (eTable 7 in the Supplement). Measures were matched at the specific time (ie, acute biomarker with acute clinical outcome) and across times (ie, acute biomarker with 24- to 48-hour clinical outcome).

Overall, Pearson correlations with clinical outcomes were small to moderate (*r*≤0.4 for all outcomes). Only the association between acute GFAP level and 24- to 48-hour BESS score was statistically significant among cadets in the concussion group (*r* = 0.36; *P* = .049).

## Discussion

To our knowledge, this study is the first to examine combative training–associated concussions among MSA cadets using longitudinal measures of multiple protein biomarkers in blood. Notably, the control group for this investigation consisted of other cadets undergoing the same type of combative training exercises as those with concussions and thus constituted an active contact-control group, helping to account for the separate outcomes associated with combative training exercises for biomarker changes, including repetitive impacts without clinical symptoms. Although these findings are newly described in the cohort of combative training–associated concussions among military cadets, acute increases in GFAP and UCH-L1 levels have also been observed in individuals with sport-associated concussions^21^ and mild to moderate TBI who presented to the emergency department.^10^ These data suggest that GFAP and UCH-L1 biomarkers have potential for use in future studies of concussion in the combative training setting, with UCH-L1 having a relatively early period of detection and GFAP having longer-lasting increases.

After these injuries, MSA cadets experienced acute clinical changes, as measured by increased SCAT-3 symptom severity scores, increased BESS scores, and decreased SAC scores with recovery over time. The BSI-18 global severity index score was high at baseline and at the acute postinjury point, but it also indicated recovery with time. Notably, Pearson correlations between clinical measures and acute or 24- to 48-hour postinjury biomarkers were not robust. This finding suggests the potential utility of objective biomarkers to help identify injury in cadets with few symptoms or clinical signs.

Neurofilament light chain levels indicated persistent differences between cadets in the concussion and contact-control groups; these differences are interesting but distinct from previous studies of NF-L levels after concussion and suggest that NF-L levels may indicate ongoing axonal injury after TBI. In boxers, NF-L levels in cerebrospinal fluid have been found to peak at 15 days and normalize after 3 to 9 months.^22,23^ Although no increases in NF-L levels were observed in a study of soccer players after a heading training session,^24^ increased levels of NF-L were found in athletes playing collision sports, such as American football^25^ and professional Swedish ice hockey.^14^ This study provides additional support for NF-L as a subacute concussion biomarker, with potential value even days after injury.

Also notable is the finding that increases in GFAP and NF-L levels persisted beyond the point of symptom resolution. For GFAP levels, statistically significant group increases were observed, even at the asymptomatic postinjury point. For NF-L levels, group differences were found at all postconcussion assessment points. These findings suggest the probability of incomplete biological recovery at the time of clinical recovery. Other studies and systematic reviews^26,27^ have suggested that TBI pathophysiologic changes may outlast clinical signs and symptoms, raising the possibility that, in addition to diagnosis, biomarkers may help to identify when an individual has recovered sufficiently to return to full duty and activity.

### Limitations

This study has several limitations. The cohorts overall are relatively modest in size. Some group differences were present at baseline for SCAT-3 symptom severity and BSI-18 global severity. It is possible that the age difference between cadets in the concussion and contact-control groups partially explains this finding, with younger cadets in the contact-control group more likely than older cadets in the contact-control group to be experiencing the initial stress of the academy and basic training. These differences in baseline measures may have consequences for the association between clinical and biomarker findings; however, they would not necessarily interfere with the objective assay measures of individual biomarkers.

Another consideration is that the number of cadets in the concussion group who also had baseline blood sample collection for biomarkers was relatively small, producing higher baseline variance among those with concussions and potentially obscuring subtle within-group differences between baseline and the acute postinjury point. The sensitivity analysis indicated similar acute and 24- to 48-hour SCAT-3 symptom severity scores among cadets with and without blood samples at the acute postinjury point, reducing concern about bias among cadets for whom blood samples were available.

Overall, the receiver operating characteristic analysis indicated that the addition of blood biomarkers did not provide greater discrimination between cadets in the concussion and contact-control groups than did SCAT-3 scores at the acute or 24- to 48-hour postinjury points. However, the patterns of biomarker change reported after combative training concussions in the present study were consistent with those found in studies of concussion and TBI in other settings.^10,21^ These patterns may better inform future work directed at translating these and other blood biomarker findings to develop the clinical end points (eg, positive and negative predictive values) that will be necessary to determine clinical utility. Examining biomarkers in distinct military subgroups may also be important to better delineate clinical utility in combat and combat training settings.

## Conclusions

This study found that blood levels of biomarkers for mild TBI and concussion are high after concussions incurred during combative training among MSA cadets. In particular, the GFAP and UCH-L1 biomarkers indicate acute increases with UCH-L1 levels decreasing by 24 to 48 hours after injury and GFAP increases persisting for several days. These biomarkers also had different associations with clinical measures of recovery. Neurofilament light chain levels were generally higher in the concussion group compared with the contact-control group, suggesting that the NF-L biomarker may be measured more remotely after a concussion, although its temporal pattern in the concussion group compared with the contact-control group warrants further investigation. Blood-based TBI biomarkers have substantial potential to inform our understanding of pathophysiological changes after mild TBI and concussion among military personnel. Future associations with long-term outcomes, impact data, and advanced neuroimaging findings are all opportunities to better delineate the roles of these biomarkers.

## Notes

Supplement. **eTable 1.** Clinical Outcome Assessment Characteristics for Concussed and Control Cadets at Baseline and Follow-Up Times **eTable 2.** Within-Subjects Comparisons of Clinical Outcome Assessments in Concussed and Control Cadets **eTable 3.** Between-Subjects Comparisons of Clinical Outcome Assessments in Concussed and Control Cadets **eTable 4.** Biomarker Characteristics for Concussed and Control Cadets at Baseline and Follow-Up Times **eTable 5.** Within-Subjects Comparisons of Biomarkers in Concussed Cadets **eTable 6.** Between-Subjects Comparisons of Biomarkers in Concussed and Control Cadets **eTable 7.** Biomarker Correlations With Clinical Outcomes **eFigure.** Baseline and Postinjury Biomarker Levels in Concussed and Control Cadets
