# Supplementary material for: Assessment of Blood Biomarker Profile After Acute Concussion During Combative Training Among US Military Cadets: A Prospective Study From the NCAA and US Department of Defense CARE Consortium
Source: JAMA Netw Open. 2021 Feb 22;4(2):e2037731. doi: 10.1001/jamanetworkopen.2020.37731 (PMC7900866; doi:10.1001/jamanetworkopen.2020.37731)
Supplement: Supplement. — eTable 1. Clinical Outcome Assessment Characteristics for Concussed and Control Cadets at Baseline and Follow-Up Times eTable 2. Within-Subjects Comparisons of Clinical Outcome Assessments in Concussed and Control Cadets eTable 3. Between-Subjects Comparisons of Clinical Outcome Assessments in Concussed and Control Cadets eTable 4. Biomarker Characteristics for Concussed and Control Cadets at Baseline and Follow-Up Times eTable 5. Within-Subjects Comparisons of Biomarkers in Concussed Cadets eTable 6. Between-Subjects Comparisons of Biomarkers in Concussed and Control Cadets eTable 7. Biomarker Correlations With Clinical Outcomes eFigure. Baseline and Postinjury Biomarker Levels in Concussed and Control Cadets [file jamanetwopen-e2037731-s001.pdf]

Supplementary Online Content

Giza CC, McCrea M, Huber D, et al; CARE Consortium Investigators. Assessment of blood biomarker profile after acute concussion during combative training among US military cadets: a prospective study from the NCAA and US Department of Defense CARE Consortium. *JAMA Netw Open*. 2021;4(2):e2037731. doi:10.1001/jamanetworkopen.2020.37731

eTable 1. Clinical Outcome Assessment Characteristics for Concussed and Control Cadets at Baseline and Follow-Up Times

eTable 2. Within-Subjects Comparisons of Clinical Outcome Assessments in Concussed and Control Cadets

eTable 3. Between-Subjects Comparisons of Clinical Outcome Assessments in Concussed and Control Cadets

eTable 4. Biomarker Characteristics for Concussed and Control Cadets at Baseline and Follow-Up Times

eTable 5. Within-Subjects Comparisons of Biomarkers in Concussed Cadets

eTable 6. Between-Subjects Comparisons of Biomarkers in Concussed and Control Cadets

eTable 7. Biomarker Correlations With Clinical Outcomes

eFigure. Baseline and Postinjury Biomarker Levels in Concussed and Control Cadets

This supplementary material has been provided by the authors to give readers additional information about their work.

© 2021 Giza CC et al. *JAMA Network Open*.

eTable 1. Clinical Outcome Assessment Characteristics for Concussed and Control Cadets at Baseline and Follow-Up Times

		Concussed			Cadet Control		
		n	Mean	SD	n	Mean	SD
Baseline	SCAT	65	11.00	15.02	35	4.11	7.86
	SAC	62	28.19	1.60	35	28.54	1.48
	BESS	62	17.03	6.43	35	14.63	6.41
	BSI-18	65	4.98	9.12	35	1.37	2.66
Acute Post-injury	SCAT	45	28.18	18.46	35	1.83	3.04
	SAC	46	27.35	2.04	36	28.19	1.45
	BESS	44	19.80	8.78	36	13.36	7.26
24-48 Hours Post-injury	SCAT	61	24.21	20.39	34	1.71	2.39
	SAC	60	27.73	1.61	34	28.38	1.30
	BESS	60	18.15	7.96	34	11.76	7.06
	BSI-18	61	4.13	4.48	34	0.74	1.56
Asymptomatic	SCAT	65	0.43	1.93	35	1.17	2.04
	SAC	65	28.91	1.22	35	28.69	1.43
	BESS	65	14.25	4.75	35	10.43	6.32
	BSI-18	62	1.10	2.77	35	0.37	1.31
7 Days Post-RTA	SCAT	55	0.22	0.71	35	0.40	0.85
	SAC	55	29.27	1.18	35	29.46	0.74
	BESS	54	12.19	5.84	35	10.57	6.20
	BSI-18	55	1.00	7.42	35	0.09	0.37

SCAT, Sport Concussion Assessment Tool; SAC, Standardized Assessment of Concussion; BESS, Balance Error Scoring System; BSI-18, Brief Symptom Inventory; SD, standard deviation; RTA, return-to-activity
Clinical outcome assessments: SCAT symptom severity score, SAC total score, BESS total errors, and BSI-18 Global Severity Index
BSI-18 was not administered at the acute post-injury time.

© 2021 Giza CC et al. *JAMA Network Open*.

eTable 2. Within-Subjects Comparisons of Clinical Outcome Assessments in Concussed and Control Cadets

			Concussed		Cadet Control	
	Time point Comparison		Mean Difference	*P*	Mean Difference	*P*
SCAT Symptom Severity Score	Baseline vs.	Acute Post-Injury	-17.82	**<.001**	NS	
		24-48 Hours Post-Injury	-12.90	**<.001**		
		Asymptomatic	10.62	**<.001**		
		7 Days Post-RTA	10.42	**<.001**		
	Acute Post-Injury vs.	24-48 Hours Post-Injury	4.92	.128		
		Asymptomatic	28.44	**<.001**		
		7 Days Post-RTA	28.24	**<.001**		
	24-48 Hours Post-Injury vs.	Asymptomatic	23.52	**<.001**		
		7 Days Post-RTA	23.32	**<.001**		
	Asymptomatic vs.	7 Days Post-RTA	-0.198	>.999		
SAC Total Score	Baseline vs.	Acute Post-Injury	0.82	**.010**	0.35	>.999
		24-48 Hours Post-Injury	0.45	.487	0.14	>.999
		Asymptomatic	-0.75	**.008**	-0.15	>.999
		7 Days Post-RTA	-1.11	**<.001**	-0.92	**.023**
	Acute Post-Injury vs.	24-48 Hours Post-Injury	-0.37	>.999	-0.21	>.999
		Asymptomatic	-1.58	**<.001**	-0.50	.947
		7 Days Post-RTA	-1.93	**<.001**	-1.27	**<.001**
	24-48 Hours Post-Injury vs.	Asymptomatic	-1.21	**<.001**	-0.29	>.999
		7 Days Post-RTA	-1.56	**<.001**	-1.06	**.005**
	Asymptomatic vs.	7 Days Post-RTA	-0.35	>.999	-0.77	.104
BESS Total Errors	Baseline vs.	Acute Post-Injury	-2.58	.109	1.27	>.999
		24-48 Hours Post-Injury	-0.90	>.999	2.92	.165
		Asymptomatic	2.81	**.018**	4.30	**.004**
		7 Days Post-RTA	4.94	**<.001**	4.15	**.006**
	Acute Post-Injury vs.	24-48 Hours Post-Injury	1.68	>.999	1.65	>.999
		Asymptomatic	5.39	**<.001**	3.03	.114
		7 Days Post-RTA	7.52	**<.001**	2.89	.159
	24-48 Hours Post-Injury vs.	Asymptomatic	3.72	**<.001**	1.38	>.999
		7 Days Post-RTA	5.84	**<.001**	1.24	>.999
	Asymptomatic vs.	7 Days Post-RTA	2.13	.232	-0.14	>.999
BSI-18 Global Severity Index	Baseline vs.	24-48 Hours Post-Injury	NS		NS	
		Asymptomatic				
		7 Days Post-RTA				
	24-48 Hours Post-Injury vs.	Asymptomatic				
		7 Days Post-RTA				
	Asymptomatic vs.	7 Days Post-RTA				

SCAT, Sport Concussion Assessment Tool; SAC, Standardized Assessment of Concussion; BESS, Balance Error Scoring System; BSI-18, Brief Symptom; Inventory; NS, Non-significant simple main effect; RTA, return-to-activity.
Results are reported only for comparisons with significant simple main effects. BSI-18 was not administered at the acute post-injury time. All *P*-values are corrected for multiple comparisons (Bonferroni).

© 2021 Giza CC et al. *JAMA Network Open*.

eTable 3. Between-Subjects Comparisons of Clinical Outcome Assessments in Concussed and Control Cadets

	Baseline		Acute Post-Injury		24-48 Hours Post-Injury		Asymptomatic		7 Days Post-RTA	
	Mean Difference	*P*	Mean Difference	*P*	Mean Difference	*P*	Mean Difference	*P*	Mean Difference	*P*
SCAT	6.92	**.004**	27.06	**<.001**	22.28	**<.001**	-0.714	.763	0.255	.916
SAC	-0.37	.224	-0.85	**.009**	-0.69	**.027**	0.230	.451	-0.188	.550
BESS	2.37	.092	6.22	**<.001**	6.19	**<.001**	3.86	**.006**	1.59	.268
BSI-18	NS									

SCAT, Sport Concussion Assessment Tool; SAC, Standardized Assessment of Concussion; BESS, Balance Error Scoring System; BSI-18, Brief Symptom Inventory; NS, Non-significant simple main effect; RTA, return-to-activity.
Results are reported only for comparisons with significant simple main effects. BSI-18 was not administered at the acute post-injury time. All *P*-values are corrected for multiple comparisons (Bonferroni).

© 2021 Giza CC et al. *JAMA Network Open*.

eTable 4. Biomarker Characteristics for Concussed and Control Cadets at Baseline and Follow-Up Times

		Concussed					Cadet Control				
		n	Mean	SD	Median	IQR	n	Mean	SD	Median	IQR
Baseline	GFAP	10	53.74	19.81	53.99	[36.2-72.22]	36	55.59	17.62	54.5	[41.94-69.14]
	UCH-L1	7	6.24	4.24	6.18	[1.29-9.78]	28	9.07	7.55	6.51	[3.63-11.75]
	NF-L	10	7.3	3.05	6.73	[5.68-9.14]	36	6.54	2.81	5.97	[4.18-8.77]
	tau	8	0.8	0.64	0.75	[0.15-1.37]	35	1.11	1.03	0.77	[0.39-1.3]
Acute Post-injury	GFAP	37	81.72	33.15	77.44	[58-93.73]	35	56.53	21.39	50.39	[42.53-77.42]
	UCH-L1	28	19.62	22.71	13.16	[7.03-22.97]	24	8.26	8.28	6.17	[3.13-11.46]
	NF-L	37	7.6	3.23	6.83	[5.34-9.62]	35	5.39	2.51	5.11	[3.42-6.19]
	tau	37	1.43	1.73	0.99	[0.47-1.91]	31	1.15	0.77	0.97	[0.63-1.67]
24-48 Hours Post-injury	GFAP	57	75.98	32.3	68.77	[48.81-94.09]	32	61.14	24.11	56.17	[46.55-76.11]
	UCH-L1	37	12.09	11.2	9.51	[3.45-16.68]	21	7.48	7.26	4.45	[3.12-10.42]
	NF-L	57	7.96	7.06	6.62	[4.93-9.63]	32	5.56	2.35	4.75	[3.83-7.18]
	tau	52	0.98	0.62	0.81	[0.58-1.22]	29	1.06	0.75	0.98	[0.51-1.33]
Asymptomatic	GFAP	62	73.16	29.57	67.95	[53.73-85.27]	34	57.64	22.67	50.27	[42.42-69.36]
	UCH-L1	42	12.34	11.82	7.9	[3.34-18.89]	23	8.24	6.15	8.59	[3.29-11.88]
	NF-L	62	9.19	12.51	6.71	[5.25-9.5]	34	5.52	2.49	4.98	[3.59-6.54]
	tau	61	1.03	0.58	0.93	[0.56-1.59]	34	1.03	0.76	0.73	[0.53-1.41]
7 Days Post-RTA	GFAP	53	69.94	29.35	61.1	[50.58-89.23]	33	58.67	19.67	54.45	[42.58-68.1]
	UCH-L1	39	9.4	8.54	7.12	[3.3-11.8]	24	6.18	4.95	5.9	[1.66-8.37]
	NF-L	53	8.63	7.97	6.39	[5.25-9.19]	33	5.87	2.86	5.25	[3.78-7.22]
	tau	53	0.86	0.56	0.78	[0.43-1.22]	30	0.98	0.84	0.76	[0.48-1.33]

GFAP, glial fibrillary acidic protein; UCH-L1, ubiquitin C-terminal hydrolase-L1; NF-L, neurofilament light; SD, standard deviation; IQR, interquartile range; RTA, return-to-activity

Units represent pg/mL for all biomarkers.

GFAP: limit of detection (LOD) = 0.221 pg/mL, lower limit of quantification (LLOQ) = 0.467 pg/mL, upper limit of quantification (ULOQ) = 850 pg/mL; UCH-L1: LOD = 1.74 pg/mL, LLOQ = 5.45 pg/mL, ULOQ = 8855 pg/mL; NF-L: LOD = 0.104 pg/mL, LLOQ = 0.241 pg/mL, ULOQ = 429 pg/mL; tau: LOD = 0.024 pg/mL, LLOQ = 0.053 pg/mL, ULOQ = 84.9 pg/mL

© 2021 Giza CC et al. *JAMA Network Open*.

eTable 5. Within-Subjects Comparisons of Biomarkers in Concussed Cadets

			Concussed	
	Time point Comparison		Mean Difference[a]	*P*
GFAP	Baseline vs.	Acute Post-Injury	-0.16	.245
		24-48 Hours Post-Injury	-0.09	>.999
		Asymptomatic	-0.03	>.999
		7 Days Post-RTA	0.02	>.999
	Acute Post-Injury vs.	24-48 Hours Post-Injury	0.07	.762
		Asymptomatic	0.13	**.013**
		7 Days Post-RTA	0.18	**<.001**
	24-48 Hours Post-Injury vs.	Asymptomatic	0.06	.928
		7 Days Post-RTA	0.18	**.021**
	Asymptomatic vs.	7 Days Post-RTA	0.05	>.999
UCH-L1	Baseline vs.	Acute Post-Injury	-0.93	.088
		24-48 Hours Post-Injury	-0.16	>.999
		Asymptomatic	-0.30	>.999
		7 Days Post-RTA	-0.06	>.999
	Acute Post-Injury vs.	24-48 Hours Post-Injury	0.77	**.001**
		Asymptomatic	0.62	**.014**
		7 Days Post-RTA	0.87	**<.001**
	24-48 Hours Post-Injury vs.	Asymptomatic	-0.16	>.999
		7 Days Post-RTA	0.10	>.999
	Asymptomatic vs.	7 Days Post-RTA	0.25	>.999
NF-L	Baseline vs.	Acute Post-Injury	NS	
		24-48 Hours Post-Injury		
		Asymptomatic		
		7 Days Post-RTA		
	Acute Post-Injury vs.	24-48 Hours Post-Injury		
		Asymptomatic		
		7 Days Post-RTA		
	24-48 Hours Post-Injury vs.	Asymptomatic		
		7 Days Post-RTA		
	Asymptomatic vs.	7 Days Post-RTA		
tau	Baseline vs.	Acute Post-Injury	NS	
		24-48 Hours Post-Injury		
		Asymptomatic		
		7 Days Post-RTA		
	Acute Post-Injury vs.	24-48 Hours Post-Injury		
		Asymptomatic		
		7 Days Post-RTA		
	24-48 Hours Post-Injury vs.	Asymptomatic		
		7 Days Post-RTA		
	Asymptomatic vs.	7 Days Post-RTA		

[a]Mean differences of natural log-transformed biomarker values. NS, Non-significant simple main effect: RTA, return-to-activity.
Results are reported only for comparisons with significant simple main effects. All *P*-values are corrected for multiple comparisons (Bonferroni).

© 2021 Giza CC et al. *JAMA Network Open*.

eTable 6. Between-Subjects Comparisons of Biomarkers in Concussed and Control Cadets

	Acute Post-Injury		24-48 Hours Post-Injury		Asymptomatic	
	Mean Difference[a]	*P*	Mean Difference	*P*	Mean Difference	*P*
GFAP	0.34	**<.001**	0.22	**.007**	0.21	**.010**
UCH-L1	0.97	**<.001**	0.31	.249	0.28	.272
NF-L	NS					
tau	NS					

[a]Mean differences of natural log-transformed biomarker values. NS, Non-significant simple main effect; RTA, return-to-activity.
Results are reported only for comparisons with significant simple main effects. Mean differences All *P*-values are corrected for multiple comparisons (Bonferroni).

© 2021 Giza CC et al. *JAMA Network Open*.

eTable 7. Biomarker Correlations With Clinical Outcomes

**Biomarker and Clinical Outcome Matched at Timepoint**									
		Concussed				Contact Control			
		GFAP	UCH-L1	NF-L	tau	GFAP	UCH-L1	NF-L	tau
Acute	SCAT-3 Symptom Severity	.114	.079	.176	.069	.047	.009	-.240	-.062
	BESS Total Score	-.205	.270	-.056	.158	-.172	-.073	-.030	.036
	SAC Total Score	.055	.163	.224	.228	.101	.124	.151	.113
24-48 Hours	SCAT-3 Symptom Severity	.028	.044	-.096	.200	.115	.402	-.201	-.266
	BESS Total Score	-.006	-.105	-.103	.115	-.199	-.302	-.116	.085
	SAC Total Score	-.058	-.033	.246	-.022	-.074	-.154	.096	.011
	BSI-18 Global Severity Index	.033	.169	.004	.217	.077	.248	-.182	.005
**Acute Post-Injury Biomarker Matched with 24-48 Hour Clinical Outcome**									
		GFAP	UCH-L1	NF-L	tau	GFAP	UCH-L1	NF-L	tau
24-48 Hours	SCAT-3 Symptom Severity	.135	.126	.165	.152	.162	.072	-.022	-.293
	BESS Total score	**.357***	.132	.302	.022	-.050	-.267	-.057	-.044
	SAC Total Score	.133	.195	.213	-.011	-.191	-.061	.022	.075
	BSI-18 Global Severity Index	.302	.383	.178	.345	.152	.055	-.071	-.035

Note. All biomarker values were natural log-transformed.
SCAT-3, Sport Concussion Assessment Tool, 3rd Edition; BESS, Balance Error Scoring System; Standardized Assessment of Concussion; BSI-18, Brief Symptom Inventory.
* $p < .05$

© 2021 Giza CC et al. *JAMA Network Open*.

eFigure. Baseline and Postinjury Biomarker Levels in Concussed and Control Cadets

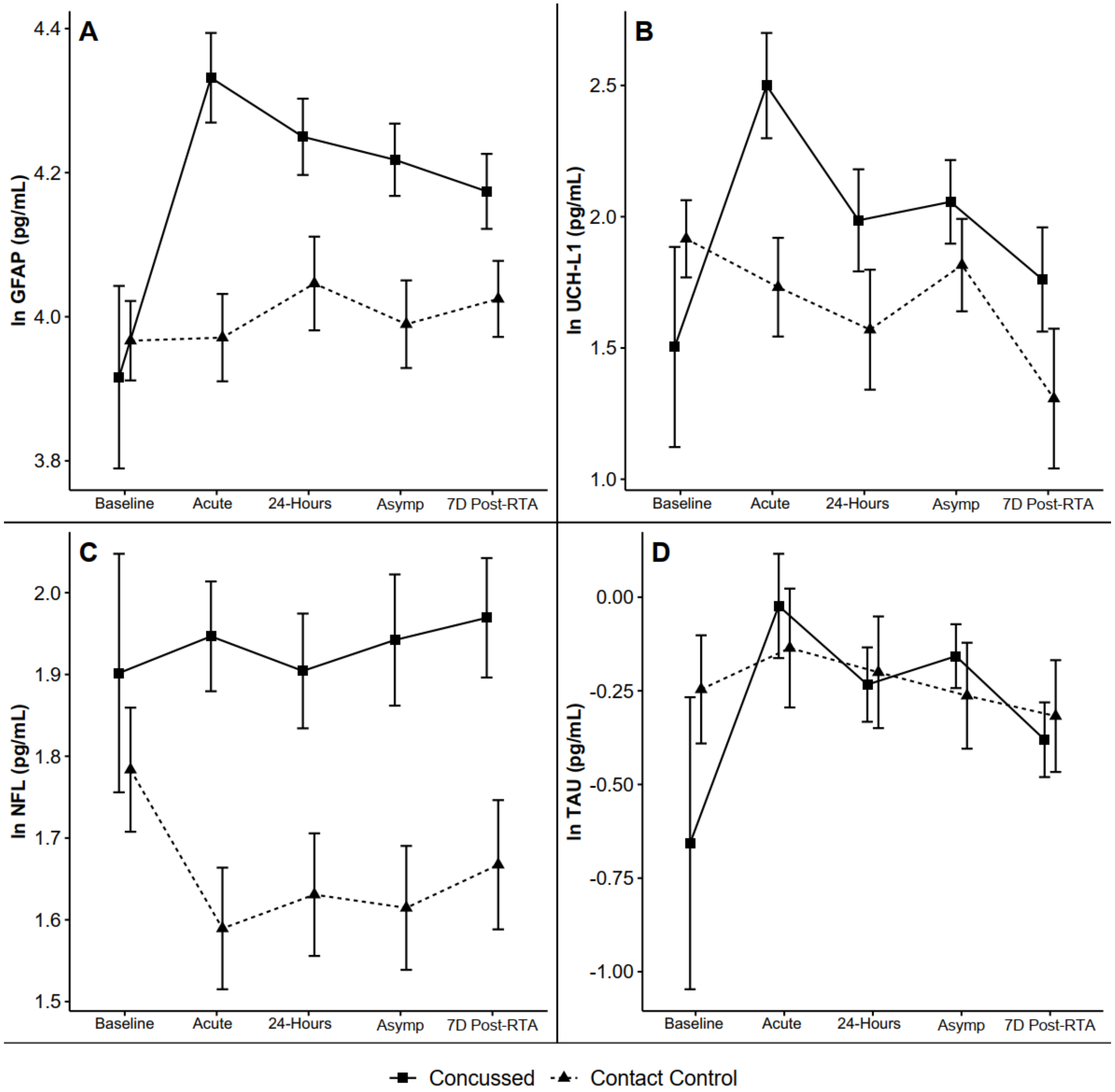

GFAP: glial fibrillary acidic protein; UCH-L1: ubiquitin C-terminal hydrolase-L1; NF-L: neurofilament light; Asymp=point at which athlete reported being asymptomatic and return to play protocol was initiated; 7D Post-RTA=7 days after unrestricted return-to-activity.

Biomarker levels represent natural log transformed scale

Error bars represent +/- one standard error

© 2021 Giza CC et al. *JAMA Network Open*.
